# The effects of enteral tube feeding on nutrition, survival, and quality of life outcomes in advanced upper gastrointestinal cancers: a systematic literature review

**DOI:** 10.1007/s00520-025-09263-6

**Published:** 2025-02-26

**Authors:** Adriana Mannino, Caroline Lasry, Julia Kuypers, Terry P. Haines, Daniel Croagh, Lauren Hanna, Kate Furness

**Affiliations:** 1 Department of Food, Nutrition and Dietetics, School Allied Health, Human Services and Sport, La Trobe University, Plenty Rd & Kingsbury Dr, Bundoora, Melbourne, Victoria 3086 Australia; 2 Nutrition and Dietetics, St Vincents Hospital, Fitzroy, Melbourne, Victoria 3065 Australia; 3 School of Primary and Allied Health Care &, Faculty of Medicine, Nursing and Health Sciences, National Centre for Healthy Ageing, Monash University, Moorooduc Highway, Frankston, Victoria 3199 Australia; 4 Department of Surgery, Faculty of Medicine, Nursing and Health Sciences, Monash University, Clayton, Victoria 3168 Australia; 5 Department of Nutrition, Dietetics and Food, Monash University, Clayton, Victoria 3168 Australia

**Keywords:** Cancer, Enteral nutrition, Nutrition, Oncology, Quality of life, Upper gastrointestinal cancer

## Abstract

**Purpose:**

Despite guidelines, enteral tube feeding is not routinely provided to advanced upper gastrointestinal (UGI) cancer patients who cannot consume adequate nutrition and who have an expected survival of at least 3 months. This review examined its effect on nutrition status, survival, and quality of life (QOL) in these patients.

**Methods:**

Five databases (CINAHL, Cochrane, Embase, Ovid, Web of Science) were searched for original research on nutrition, survival, and/or QOL outcomes in adults with inoperable UGI cancers receiving enteral tube feeding. Quality was assessed using the Academy of Nutrition and Dietetics Quality Criteria Checklist: Primary Research, and a narrative synthesis was conducted.

**Results:**

Five studies were eligible for inclusion, most participants were male (*n* = 205), with low sample sizes across all studies (*n* = 16–131). Enteral tube feeding resulted in a similar proportion of participants with weight loss above or below 5% (baseline to 12 weeks) compared to a control group [*p* > 0.05] (1 study), and a significant increase in mean lean body mass [+1.3 (± 4.0) kg, *p* = 0.01] (1 study). There was variability in survival outcomes, statistical modelling, and comparators in five studies, with subsequently contradictory results. Only one study reported on QOL. Study quality was assessed as neutral (4 studies) or negative (1 study), reflecting methodological/analytical issues across the studies.

**Conclusions:**

This systematic literature review highlights a significant knowledge gap, with no high-quality randomised controlled trial-based evidence available on enteral nutrition efficacy, limiting its use in dietetic practice in this sub-population. Despite treatment developments prolonging survival, research investigating feeding and its impact on QOL remains inadequate. Further research is needed to promote change and influence practice, policy, and guidelines, alongside high-quality intervention studies with defined nutrition outcomes, regimens, and robust statistical analyses to determine the benefits of enteral tube feeding in this vulnerable population.

**Supplementary Information:**

The online version contains supplementary material available at 10.1007/s00520-025-09263-6.

## Introduction

Cancers affecting the upper gastrointestinal (UGI) tract are amongst the leading causes of cancer-related deaths worldwide [1, 2]. The incidence of UGI cancers is growing annually, with an estimated projected combined incidence of more than 15,000 new cases of UGI cancers in Australia in 2023 [3]. In the same year, pancreatic cancer was the eighth most commonly diagnosed cancer in Australia, with liver, stomach, and oesophageal cancers amongst the top 20 [3]. Five-year survival of UGI cancers are amongst the lowest across cancer survival rates in Australia: pancreatic 12.5%; gall bladder 19.1%; liver 22.9%; oesophageal 23.7%; and stomach 37.7% [3]. Up to 85% of some UGI cancers are unresectable at presentation and hence deemed incurable and are for palliative treatment only [4].

During UGI cancer treatment, nutrition-impact symptoms such as dysphagia, steatorrhoea, anorexia, nausea, and vomiting can cause both a reduced desire to eat and maldigestion and malabsorption of nutrients. Physical obstructions caused by UGI tumours can also cause an inability to swallow and subsequent avoidance of food [5]. In addition to these factors, inflammation results in the loss of skeletal muscle mass [5], contributes to gut barrier dysfunction [6], and can further exacerbate nutrition-impact symptoms. These factors combined accelerate the development and/or severity of weight loss and malnutrition leading to cancer cachexia which is not reversible with conventional nutrition support and leads to progressive functional impairment [6]. It is estimated that 20–70% of cancer patients have some degree of malnutrition contributing to approximately 20% of cancer deaths [7]. Malnutrition, weight loss, and sarcopenia [6] are common side effects of UGI cancer and its treatments [5, 7], and are associated with an increased risk of treatment-related toxicities, shorter treatment times (lower completion rates), lower response rates, and higher mortality [8].

In addition to treatment and survival outcomes, nutritional status also influences quality of life (QOL) in cancer [8, 9], an outcome of significant importance to patients with advanced cancers [10]. A 2012 systematic review of UGI patients explored the association of appetite and weight loss with reduced QOL and found that nutritional status and weight loss may be a strong predictor of QOL, and by way of treating malnutrition, there can be a significant positive impact on a patient’s QOL [9]. However, a 2021 systematic review and meta-analysis of eleven randomised controlled trials found that dietary counselling and/or high-energy oral nutrition support had no effect on body weight during chemo(radio)therapy in a mixed cancer cohort [11]. Similarly, another systematic review of 17 studies conducted in older cancer patients reported no benefit of oral nutritional support for survival outcomes, treatment feasibility, and only limited benefit to QOL [12]. A recent randomised controlled trial [13] found that intensive remote nutrition counselling and behaviour change techniques using oral nutrition support alone were not adequate to meet nutrition requirements or impact the nutritional status in UGI cancer patients. Furthermore, it was shown that when comparing intensive remote nutrition counselling with standard care, there was no difference in QOL assessments in this study [13].

Given evidence suggests that the success of dietary counselling and oral nutrition support is limited, escalation to other forms of nutrition support may be necessary to consistently meet the nutritional requirements in the UGI population [13] to improve nutritional outcomes and QOL. Clinical practice guidelines recommend all patients receiving anti-cancer treatment with an expected survival of more than three months receive nutrition support, including enteral nutrition support [8, 14]. This is contrary to current care [15], as controversy surrounds the provision of perceived invasive nutrition support in patients with advanced cancer [16].

Palliative intent treatment does not mean end-of-life care as patients with advanced cancer often undergo anti-cancer treatments to improve symptom management, function, QOL, and survival with nutrition support; enteral and parenteral nutrition are important adjuncts to these therapies [17]. This systematic literature review aimed to investigate the effect of enteral tube feeding on markers of nutrition status, survival, and QOL in adult patients with advanced upper GI cancers.

## Methods

### Protocol and registration

This systematic literature review was conducted according to the Preferred Reporting Items for Systematic Reviews and Meta-Analyses (PRISMA) guidelines [18]. The protocol was registered with the International Prospective Register of Systematic Reviews (PROSPERO) database (CRD42023434426) on 11 July 2023.

### Eligibility criteria

Full-text, original research studies published in peer-reviewed journals from 1 January 2008 to 22 October 2023 were eligible for inclusion. This date range was decided through an initial search of the literature highlighting modern enteral nutrition practices in clinical practice. The review included studies in languages other than English with translation by machine translators [19]; articles not able to be translated into English were excluded. Table 1 presents a summary of the inclusion and exclusion criteria for the Participant, Intervention, Comparator, Outcome, and Study design concepts (PICOS) [20]. Outcomes of interest included nutrition status markers (e.g. weight, weight loss, malnutrition risk scores, malnutrition assessments), survival, and quality of life outcomes assessed using validated tools.

**Table 1:** Study inclusion and exclusion criteria according to the participant, intervention, comparator, outcome, and study design concepts (PICOS) framework [20]

	Inclusion criteria	Exclusion criteria
Participant	Adult humans over 18 years of age Studies including patients with inoperable cancer of the oesophagus, stomach, liver, bile ducts, pancreas, and small intestines (locally advanced or metastatic)	Paediatric participants Studies including non-UGI or mixed cancer cohorts where results cannot be separated for patients with upper GI cancers Studies including patients receiving definitive or curative intent treatments where results cannot be separated for patients with palliative intent
Intervention	Interventions using enteral nutrition via a feeding tube (with or without oral nutrition supplements)	Parenteral nutrition or oral nutrition supplements Studies investigating pre/postoperative nutrition interventions Studies investigating immuno-nutrition
Comparator	No comparator	
Outcome	Nutrition status markers, survival, quality of life outcomes	
Study design	Studies published in peer-reviewed journal Quantitative research studies (randomised and non-randomised control, cross-sectional and cohort studies; secondary analyses)	Qualitative studies; reviews; letters to the editor Conference abstracts

*UGI* upper gastrointestinal

### Search strategy and information sources

The literature search was conducted in five databases: CINAHL (Cumulative Index of Nursing and Allied Health Literature) via EBSCO, Cochrane Central Register of Controlled Trials, Embase, Ovid MEDLINE, and Web of Science. Truncations and Boolean Operators were applied to relevant keywords and subject headings for each database. Identical limits were applied for publication dates (> 2008 to 22 October 2023), language (English or machine translatable [19]), and age (18 years and over)). The complete search strategy for each database is presented in the Supplementary Material.

### Study selection

**Fig. 1:**
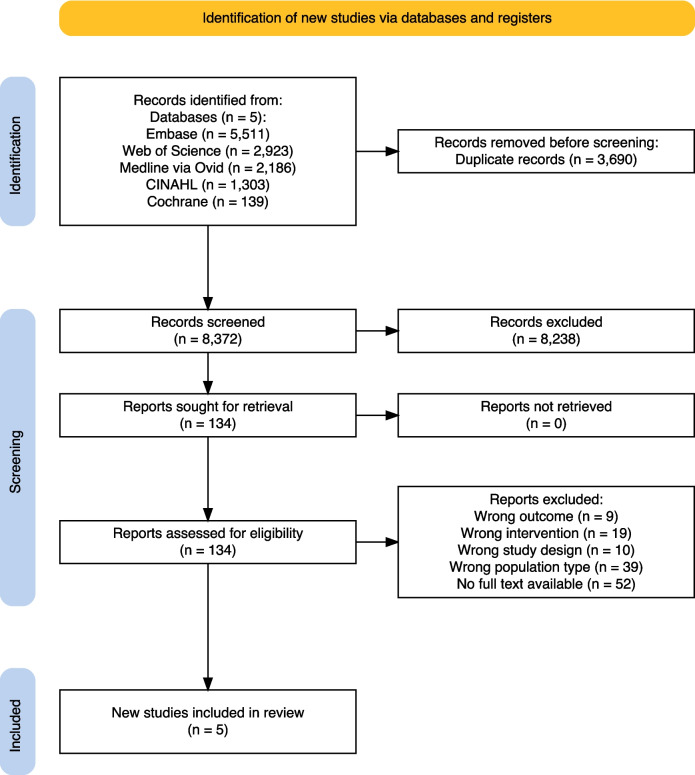
Flowchart of the search, depicting the selection process of the included studies; PRISMA diagram

A systematic search, database retrieval, and de-duplication were conducted by the first reviewer. Studies retrieved were imported to EndNote (Version 20) [21], where a process of de-duplication occurred using the Bramer method of deduplication [22]. De-duplicated studies were then imported to Covidence® [23], where additional duplications were removed. The second and third reviewers independently screened study titles and abstracts against the inclusion criteria, with the first reviewer completing a repeat screening of 20% of studies to ensure accuracy. Full texts were then independently reviewed by the second and third reviewers, and all three reviewers reached consensus for the final number of records included. The reasons for exclusion were documented and are presented in Fig. 1.

### Data extraction and data items

Data were extracted independently by the second and third reviewers using Covidence® and were subsequently exported into a Microsoft Excel Spreadsheet for analysis. Data extracted included study design, population description, cancer type/stage, number of participants, intervention type, comparator type, outcome types, and results. Consensus on data extraction was reached through discussion between all reviewers after the initial data extraction process was completed.

### Risk of bias and quality assessment in individual studies

Assessment for risk of bias was completed by the second and third reviewers independently using the Academy of Nutrition and Dietetics Quality Criteria Checklist: Primary Research [24]. Reviewers completed the checklist for each study, with two alternative reviewers resolving conflicts.

### Data synthesis and summary measures

The review presents a narrative synthesis of the results reported by the selected studies, with the use of the synthesis without meta-analysis (SWiM) in systematic reviews: reporting guideline [25]. The narratively synthesised results present feeding interventions and outcomes including body weight, BMI, body composition changes, serum albumin, survival, and objective QOL measures.

## Results

### Study selection

From the five databases searched, 12,062 studies were retrieved for screening (see Fig. 1). After a process of de-duplication whereby 3690 studies were removed, 8372 studies remained for the title and abstract screening. One-hundred and thirty-four studies proceeded to full-text review. During the full-text review, 129 were excluded due to wrong outcomes (*n* = 9), wrong intervention (*n* = 19), wrong study design (*n* = 10), wrong population type (*n* = 39), and no full-text available (*n* = 52). Four studies’ authors were emailed to seek clarification or further information, but no response was received. As a result, five studies were eligible for inclusion in this systematic literature review.

### Study characteristics

Across the five studies included in the review, the total number of participants was *n* = 233, with the number of participants ranging from *n* = 16 [26] to *n* = 131 [27]. The included studies were conducted in the USA (*n* = 2) [26, 28], Japan (*n* = 1) [27], Taiwan (*n* = 1) [29], and Portugal (*n* = 1) [30]. Study design included a retrospective study (*n* = 1) [27], non-randomised experimental study (*n* = 1) [26], retrospective cohort study (*n* = 2) [29], and retrospective matched cohort study (*n* = 1) [28]. A summary of the characteristics of the included studies is presented in Table 2.

**Table 2:** Characteristics of included studies, and key outcomes describing the impact of enteral nutrition on nutritional status and survival in people with advanced upper gastrointestinal cancer

Author Year Country Study design	Cancer type Cancer stage	Sample size *n* (% male)	Age, yrs (mean ± SD)	Enteral tube feeding intervention Follow-up timepoints	Comparator	Outcomes				
						Weight	BMI	Body composition	Albumin	Survival
Gresham et al. 2021 USA Non-randomised experimental study	Pancreatic Advanced or locally advanced	16 (43.8)	67 ± 9.3	Supplemental jejunal or gastro-jejunal feeding with semi-elemental formula Day 28, Day 56, Day 84 (28-day cycles)	-	Mean weight change^a^ + 1.3 (± 5.8) kg, *p* > 0.05 *n* = 4 weight stable, *n* = 6 weight increase, *n* = 6 weight loss	Mean BMI change^a^ + 0.6 (± 1.7) kg/m^2^, *p* > 0.05	Mean lean body mass change^a^ +1.3 (± 4.0) kg, *p* = 0.01 Mean fat mass change^a^ −0.6 (± 2.8) kg, *p* > 0.05	-	Median survival (days)^b^: 316 No difference in survival between participants with weight loss (*n* = 6) vs weight stable/gain (*n* = 10), HR 1.31, 95% CI 0.41–4.14, *p* = 0.64
Grilo et al. 2012 Portugal Retrospective cohort study	Oesophageal Stage III–IV	17 (100)	60.9 ± 12.8	PEG tube for comfort palliative nutrition 1 month, 3 months	-	-	Mean BMI (kg/m^2^) reported: Baseline 21.3 (± 3.5) 1-month^c^ 21.0 (± 3.1) 3-months^d^ 20.6 (± 3.0)	-	Mean albumin (g/dL) reported: Baseline 3.8 (± 0.6) 1-month^c^ 3.7 (± 0.7) 3-months^d^ 3.9 (± 0.5)	12 deaths recorded Mean survival (months): 5.9
Kakuta et al. 2019 Japan Retrospective cohort study	Oesophageal Stage II–IVb	131 (90)	Group 1: 68 (41–85)^e^ Group 2: 67 (44–91)^e^ Group 3: 64 (44–75)^e^	Group 1: stent insertion (*n* = 38) Group 2: Tube enterostomy for enteral nutrition (*n* = 65) Group 3: Palliative esophagectomy (*n* = 28) No follow up	Between-group comparison	-	-	-	-	Median survival (days)^b^: Group 1/2/3 88/208/226 *p* < 0.001 (Group 1 vs 2/3)^f^
Mitchell et al. 2017 USA Retrospective matched cohort study	Oesophageal, GOJ Metastatic	38 (94.7)	53.8 ±8.8	PEG tube insertion (*n* = 20) 4–6 weeks, 12 weeks	No PEG tube insertion (*n* = 18)	No difference in proportion of participants with weight loss above or below 5% (baseline to 12 weeks) between groups (*p* > 0.05)^f^	-	-	-	Median survival (days): PEG tube 173 (95% CI 89–387), no PEG tube 305 (95% CI 209–655), no significant difference after Bonferroni correction (corrected *p*-value not reported)^f^ Oesophageal cancer subgroup analysis: Overall survival shorter in PEG group 172 days (95% CI 84–297, *n* = 16) compared to no PEG 449 days (95% CI 219–729 *n* = 8), *p* = 0.0009^f,g^
Yang et al. 2015 Taiwan Retrospective cohort study	Oesophageal IIIc and IV	31 (87.1)	Group 1: 58.0 ± 9.4 Group 2: 72.6 ± 14.1 Group 3: 61.4 ± 7.8	Group 1: NG tube for enteral nutrition (*n* = 12) (second treatment offered, if patient refused stent) Group 2: Oesophageal stent (*n* = 10) (first treatment offered) Group 3: Supportive palliative medical care with fluid hydration and minimal oral intake (*n* = 9) (if patient unable to have stent or NG tube) Follow-up at 3–4 weeks (albumin)	Between-group comparison	-	-	-	Albumin decreased from baseline to 3–4 weeks in Group 3 only (supportive palliative care)	Longer survival for Group Group 1 (median 122 days, HR 0.09, 95% CI 0.02–0.36) and Group 2 (median 133 days, HR 0.16, 95% CI 0.03–0.84), compared to Group 3 (adjusted for age, fistula and dysphagia score)

*CI* confidence interval, *HR* hazard ratio, *NG* nasogastric tube PEG percutaneous endoscopic gastrostomy

^a^Baseline to end of intervention (84 days)

^b^Interquartile range not reported

^c^*n* = 15

^d^*n* = 10

^e^Median (range)

^f^Univariate analysis only

^g^Reported *p*-value before Bonferroni correction, p-value reported to be statistically significant after Bonferroni correction, however not reported

Most participants across all studies were male (*n* = 205; 88.0%), with only one study having the majority female (56.2 %) [26]. Participants were aged between 54 and 73 years. Of the five studies, three included only oesophageal cancer [27, 29, 30], one included pancreatic cancer [26], and one included both oesophageal and gastro-oesophageal cancer [28]. One study included participants with metastatic cancer only [28] all other studies included a combination of advanced cancers (stages III–IV).

### Enteral tube feeding interventions

Enteral nutrition support was delivered via percutaneous endoscopic gastrostomy tube in two studies [30, 28], nasogastric tube in one study [29], and gastric or jejunal tube enterostomy in two studies [26, 27]. The effect of enteral tube feeding interventions was assessed at follow-up time points ranging from 3–4 weeks [29] to 84 days [26]; Kakuta *et al.* reported the censorship date for a survival analysis. The study by Gresham *et al.* [26] was the only study to provide detail regarding the intervention, including the formula used (Peptamen 1.5) and the method for determining dosing of supplementary enteral tube feeding required for each participant, where oral intake assessed via 24-h recall was compared to estimated energy requirements using the Mifflin St Jeor equation with a stress factor of 1.5 [31]. The four remaining studies did not report on how the enteral feeding tube was used to administer nutrition to participants. Three studies provided detail of the method of enteral tube insertion [27, 29, 30]; in one study, whilst enteral energy intake was reported, there was no identification of whether oral intake also occurred and if total energy intake met nutritional requirements [29].

### Effect of enteral tube feeding on nutrition outcome measures

Nutrition outcomes were reported by four studies [26, 28–30], which reported varied measures, including weight, BMI, body composition changes, and serum albumin (presented in Table 2). Gresham *et al.* [26] demonstrated that supplemental jejunal or gastro-jejunal tube feeding resulted in weight maintenance in participants with pancreatic cancer, with non-significant differences in changes in weight and BMI from baseline to Day 84. Grilo *et al.* [30] found a small decrease in mean BMI from baseline to 3 months in participants with oesophageal cancer receiving comfort palliative nutrition via PEG tube, however, the significance of the decrease was not reported. In the study by Mitchell *et al.* [28], there was no difference in the proportion of participants with weight loss of over 5% in those with or without a PEG tube for enteral nutrition. Body composition was assessed in the study by Gresham *et al*. only [26], with significant increase in lean body mass and no change in fat mass observed in pancreatic cancer at cycle 3 of chemotherapy. Two studies measured albumin as an indicator of nutrition status [29, 30], which is a historically used nutrition biomarker now understood to reflect inflammation rather than nutrition [32]; only one study reported on albumin levels in those receiving an enteral tube feeding intervention, where albumin remained stable from baseline to 3-months [30].

### Effect of enteral tube feeding on overall survival

Survival was reported as an outcome in all five studies, with varying follow-up periods and methods of analysis. Yang et al. [29] found significantly longer mean survival in participants receiving enteral nutrition or post-insertion of an oesophageal stent, compared to those who received supportive palliative care only in a multivariate analysis adjusting for age, dysphagia, and presence of fistula; no survival difference between the enteral nutrition and stent groups was observed. Similarly, Kakuta et al. [27] found a significantly longer median survival time in participants who received either enteral nutrition (EN) or a palliative esophagectomy (PE) compared to insertion of self-expandable metallic stent (SEMS) in an unadjusted Kaplan-Meier analysis, with no significant difference in median survival time between EN and PE groups [27]. The survival analyses by Mitchell et al. [28] in oesophageal and gastro-oesophageal junction cancer were also unadjusted, with no significant difference in overall survival observed (after Bonferroni correction) between participants with or without a PEG tube for enteral nutrition. In a subgroup analysis of participants with oesophageal cancer only, median survival was significantly shorter in those with a PEG tube compared to the non-PEG group. Gresham et al. [26] explored overall survival in relation to weight stability and found no statistically significant difference between those with stable weight or weight gain (*n* = 10) compared to those who lost weight (*n* = 6) in a multivariate analysis (other variables in the model not reported). Grilo et al. [30] simply reported that 12 deaths occurred during the study period, with a mean survival time post-PEG insertion of 5.9 months.

### Effect of enteral tube feeding on quality of life

There was only one study (Gresham et al. [26]) which reported on health-related quality of life (HRQOL) [26], measured using both the National Institutes of Health (NIH) Patient-Reported Outcomes Measurement Information System (PROMIS) tool [33] and the cancer-specific European Organization for Research and Treatment of Cancer (EORTC) QLQ-C30 questionnaire [34] in a non-controlled intervention trial testing the feasibility of supplemental enteral feeding via jejunal or gastro-jejunal tube in advanced pancreatic cancer. Using the PROMIS tool where lower scores indicate better HRQOL, there was a significant reduction in scores for pain interference (mean change −7.5, *p* = 0.05) and depression (mean change −10.4, *p* = 0.006) from chemotherapy Cycle 1 to Cycle 3; changes in scores for fatigue, sleep disturbance, and physical function were reported to be clinically significant but not statistically significant. Scores of the EORTC QLQ-C30 indicated statistically significant improvements in global HRQOL (mean change 12.4, *p* = 0.04), role functioning (mean change 18.2, *p* = 0.04) and reduction in appetite loss (mean change −27.5, *p* = 0.02) from Cycle 1 to Cycle 2, and in role functioning (mean change 20.1, *p* = 0.03) and reduction in appetite loss (mean change −27.4, *p* = 0.02) from Cycle 1 to Cycle 3 [26]. Changes in remaining functioning and symptom scale scores of the EORTC QLQ-C30 from baseline (Cycle 1) to follow-up at Cycles 2 and 3 were reported as clinically significant improvements but were not statistically significant.

### Risk of bias within studies/ across studies

All studies included were reviewed against the Academy of Nutrition and Dietetics Quality Criteria Checklist: Primary Research [24] (Table 3). Four of the studies were assessed as neutral [26–29], and one was assessed as negative quality [30]. Methodological strengths across the studies included defining their research question clearly, relevance of the study variables to dietetics practice, and likely areas of importance to the target population. Specific methodological limitations across the studies included a lack of random allocation and the majority (80%, *n* = 4) of the studies were retrospectively designed [27–30]. There was also an absence of blinding or controlling within all studies. Furthermore, the intervention and/or therapeutic regimens were not described in detail in many of the studies nor were there considerations for confounding or intervening factors [27–30]. The appropriateness of the statistical analysis for the study design and outcome was also unclear in most of the studies [27–30].

**Table 3:**
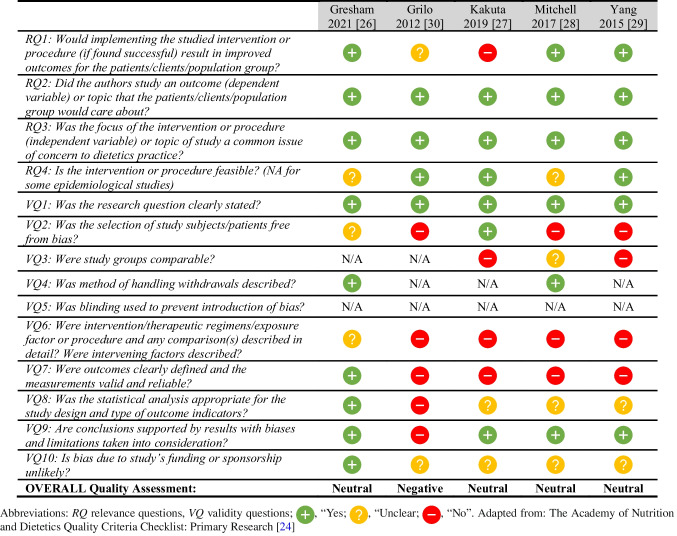
Risk of bias in individual studies, assessed using the Academy of Nutrition and Dietetics Quality Criteria. Checklist: Primary Research [24]

## Discussion

To our knowledge, this is the first systematic literature review exploring the effect of enteral tube feeding on nutrition status, survival, and quality of life in adult patients with inoperable upper GI cancers. In this review, enteral tube feeding was associated with relative longitudinal stability in nutritional status assessed by body weight and/or BMI [26, 30], with weight changes similar to controls not receiving enteral nutrition [28]. Lean body mass was reported to increase significantly in patients receiving jejunal feeding during chemotherapy for pancreatic cancer [26], and albumin levels remained stable during enteral feeding [30]. The impact of enteral tube feeding on survival outcomes was mixed, with two studies reporting survival benefit compared to other treatments such as oesophageal stent insertion [27] or comfort care [29], and another study reporting no survival differences between those with or without a PEG tube for enteral tube feeding [28]. HRQOL was reported in one study only, with some improvement in global and role functioning HRQOL scores observed as well as a decrease in symptoms such as pain, depression, and appetite loss [26]. This review addresses an important topic, however, the small number of included studies and the lack of randomised controlled trials highlight the dearth of evidence regarding the impact of enteral tube feeding on these outcomes in people with advanced upper GI cancers receiving palliative intent treatments.

To optimise nutritional status to improve survival or quality of life outcomes, a nutrition intervention needs to meet individual nutritional needs [35]. Only one of the five included studies [26] reported any details of the enteral tube feeding intervention delivered to participants, making the contribution of enteral feeding to the outcomes reported in the included studies difficult to determine. In the study by Gresham et al. [26], the intervention aimed to achieve nutritional adequacy through oral diet and supplementary enteral nutrition was clearly reported. Adequacy of oral food intake was compared to estimated nutritional requirements at the start of each chemotherapy cycle to determine the supplementary dose of enteral formula required to meet nutritional needs, although participant adherence to prescribed enteral nutrition dosing was not described. In contrast, the other included studies reported little about the intervention aside from the feeding tube type and method of feeding tube insertion. Insertion of a feeding tube alone will not influence nutritional status; rather, it is the use of a feeding tube to deliver enteral formula at a dose that is sufficient to meet individual nutritional requirements that can be considered as an intervention with the potential to improve nutrition status. The failure of most (*n* = 4, 80%) of the included studies to report how the feeding tube was used to deliver nutrition is a significant shortcoming of the small evidence base, which limits the interpretation of findings.

The rationale for enteral tube feeding differed between the included studies and may have affected the outcomes. Yang et al. [29] provided enteral tube feeding only to individuals who declined stent insertion. Similarly, Grilo et al. [30] provided enteral tube feeding to participants who could not receive a stent due to their oesophageal tumour location. The study by Kakuta et al. [27] did not report the rationale for treatment allocation so it was not clear how the clinical history or treatment plan differed for patients receiving enteral tube feeding compared with those receiving either oesophagectomy or insertion of an oesophageal stent. In that study, those who received enteral tube feeding or oesophagectomy had lower ECOG (Eastern Cooperative Oncology Group) performance status than those yet had better median overall survival than those who received a stent. Similarly, Mitchell et al. [28] did not report a rationale for participants either receiving or not receiving enteral feeding via PEG tube; it was noted that participants having PEG insertions more likely to have already started palliative treatment. In that study, there was no survival difference between groups in the overall cohort, but an unadjusted subgroup analysis in those with oesophageal cancer indicated shorter survival in the PEG group; this may have been because this group had a longer time for potential decline post-diagnosis and that they were more malnourished, with worse nutrition status and functional decline limiting survival time, however this was not discussed.

An important limitation of the evidence found in this review is that there were no studies found that accounted for the impact of chemotherapy treatment and its associated toxicity. Chemotherapy can significantly influence nutritional status, survival, and quality of life [36, 37], yet this key variable was either underreported or not considered in most studies, limiting the ability to fully understand the effects of enteral tube feeding in the context of concurrent cancer treatment.

A strength of this systematic literature review is the comprehensive search strategy developed in consultation with a specialist medical librarian in order to capture all relevant publications. This review was conducted in line with the PRISMA Statement [18], and utilised Endnote [21], and Covidence® [23] systematic review software to optimise autonomy and objectivity of the researchers during independent screening of studies [23, 38]. The low number of included studies was a limitation of this review, despite our efforts to broaden the search and protocol parameters. A reason for the exclusion of many studies during the full-text screening stage was due to the number of studies that pooled the results for curative versus inoperable UGI cancer patients. This scarcity in the literature highlights a significant gap in the published research specific to the palliative population. There was also significant heterogeneity across the included studies due to the wide range of nutrition markers and outcome measures used. Furthermore, the review highlights the overall increased risk of bias across the included studies (lack of blinding and/ or exclusion of confounding factors). Four of the five studies were retrospective studies, which by design are vulnerable to reporting bias was present regarding the intervention, as studies with favourable results are more likely to be published.

The study by Gresham et al [26] was the only study to acknowledge the role of a dietitian as part of the investigator team. It is known that physicians under-record and under-assess nutrition status despite being the most likely first point of patient contact [39], which may reflect inadequate nutrition training, inadequate knowledge of local malnutrition assessment tools, an unwillingness to take responsibility for a patient’s nutrition status, and real- or perceived time pressures [39, 40]. Dietitians are experts in the science of human nutrition, and as such, should be integral members of a multidisciplinary team caring for people with advanced cancer [41, 42]. It is the responsibility of the entire healthcare team to identify, be accountable, and manage malnutrition [43]. This requires all members to recognise the value of nutrition care and the detrimental consequences of malnutrition, to screen patients for malnutrition, to refer to dietitians for expert nutrition intervention that is rapidly implemented in consultation with the team, and then to regularly monitor and modify the tailored intervention plan where appropriate [43]. Any observed effect from an enteral tube feeding intervention in patients with advanced upper GI cancer could be potentially enhanced with dietetic input, therefore future researchers may wish to consider this design modification which has occurred in only one small study to date.

Many people with advanced cancer are provided with palliative intent chemotherapy to improve symptom control, quality of life and in some cases, survival outcomes [44–46]. Advancements in treatment modalities available to locally advanced and metastatic cancers may give people months to years of additional life [47]. Therefore, if a patient is unable to meet their nutritional requirements through oral intake, oral nutrition support, and dietetic counselling and education, artificial nutrition in the form of enteral nutrition is a feasible and recommended next step with strong expert consensus [14, 48]. Percutaneous endoscopic gastrostomy (PEG) is considered the gold standard enteral feeding route when gastric access is available or percutaneous endoscopic gastrostomy with jejunal extension (PEG-J) or surgical jejunostomy (JEJ) where gastric access is unavailable [49]. Decision-making regarding enteral nutrition should be made when there is a realistic goal and indication for ongoing medical treatment, the patient has provided informed consent, and the treating medical team agrees with its potential benefit [16, 45]. Early identification of patients who have poor oral intake, anorexia, weight loss, and are at risk of or have been diagnosed with malnutrition should be considered for enteral nutrition given that 20–30% of cancer deaths are directly attributable to malnutrition [50, 51]. A recent study of 200 patients with advanced cancer who received either usual care, enteral nutrition or oral nutrition support found that global QOL scores were not different between groups (*p* = 0.309) [52]. This provides important evidence that enteral feeding does not worsen quality of life and debunks the common myth that the perceived harm or invasiveness of enteral nutrition negatively impacts QOL [53], where QOL is the cornerstone of palliative approach therapies [52].

## Conclusion

This systematic review identified five studies which examined the use of enteral tube feeding in patients with advanced upper GI cancers. The limited number of studies found within the literature indicates a significant gap in research, with no high quality randomised controlled trial-based evidence available on enteral nutrition efficacy, limiting the ability to implement enteral nutrition pathways in dietetic clinical practice [17]. There is a considerable need for further research to promote change and influence practice, policy, and guidelines in this vulnerable population.

## Supplementary Information

Below is the link to the electronic supplementary material.

Supplementary file1 (DOCX 28 KB)

## Data Availability

No datasets were generated or analysed during the current study.
