# Supplementary material for: The effects of enteral tube feeding on nutrition, survival, and quality of life outcomes in advanced upper gastrointestinal cancers: a systematic literature review
Source: Support Care Cancer. 2025 Feb 26;33(3):223. doi: 10.1007/s00520-025-09263-6 (PMC11865217; doi:10.1007/s00520-025-09263-6)
Supplement: Supplementary file 1 — Supplementary file1 (DOCX 28 KB) [file 520_2025_9263_MOESM1_ESM.docx]

**Supplementary Tables**

**Supplementary Table 1.** Search Strategy MEDLINE (1946-) on OVID

| **Search No.** | **Search Statement** |
| --- | --- |
| 1 | **Medical Headings**  exp Pancreatic Neoplasms/ or exp Carcinoma, Pancreatic Ductal/ or exp Esophageal Neoplasms/ or exp Stomach Neoplasms/ or exp Gastrointestinal Stromal Tumors/ or exp Bile Duct Neoplasms/ or exp Gallbladder Neoplasms/ or exp Liver Neoplasms/ or exp Carcinoma, Hepatocellular/ or exp Duodenal Neoplasms/ or exp Biliary Tract Neoplasms/ or exp Splenic Neoplasms/ |
| 2 | **Key words**  (Oncolo* or cholangiocarcinoma or (duoden* adj3 (cancer*or tumo?r* or neoplasm*or carcinoma* or malignan* or adenocarcin*)) or (hepato* adj3 (cancer*or tumo?r* or neoplasm*or carcinoma* or malignan* or adenocarcin*)) or (splen* adj3 (cancer*or tumo?r* or neoplasm*or carcinoma* or malignan* or adenocarcin*)) or (gall bladder adj3 (cancer*or tumo?r* or neoplasm*or carcinoma* or malignan* or adenocarcin*)) or (liver adj3 (cancer* or tumo?r* or neoplasm* or carcinoma* or malignan*or adenocarcin*)) or (bil* adj3 (cancer* or tumo?r* or neoplasm* or carcinoma* or malignan*or adenocarcin*)) or (pancrea* adj3 (cancer*or tumo?r* or neoplasm*or carcinoma* or malignan* or adenocarcin*)) or (gastric adj3 (cancer* or tumo?r* or neoplasm* or carcinoma* or malignan*or adenocarcin*)) or (stomach adj3 (cancer*or tumo?r* or neoplasm*or carcinoma* or malignan* or adenocarcin*)) or (esophag* adj3 (cancer*or tumo?r* or neoplasm*or carcinoma* or malignan* or adenocarcin*)) or (oesophag* adj3 (cancer* or tumo?r* or neoplasm* or carcinoma* or malignan*or adenocarcin*)) or (upper gastrointestinal adj3 (cancer* or tumo?r*or neoplasm* or carcinoma* or malignan*or adenocarcin*))).mp |
| 3 | 1 OR 2 |
| 4 | **Medical headings**  exp Enteral Nutrition/ or exp Intubation, Gastrointestinal/ or exp Nutritional Support/ or dietary supplements.mp. |
| 5 | **Keywords**  ((jejun* adj3 (feed* or enteral or tube or ostomy)) or (gastr* adj3 (feed* or enteral or tube or ostomy)) or (duo* adj3 (feed* or enteral or tube)) or enteral nutrition or enteral feeding or tube feeding or enteral tube feeding or nutritional support or enteral nutrition therapy or enteral nutrition support or 'peg' or percutaneous endoscopically placed gastrostomy or nasogastric feeding tube or nasogastric tube or nasojejunal feeding tube or nasojejunal tube or jejunostomy or jejunostomy feeding or jejunostomy feeding tube or jejunal feeding or gastric feeding).mp |
| 6 | 4 OR 5 |
| 7 | **Medical Headings**  exp Nutrition Assessment/ or exp Malnutrition/ or Nutritional Status/ or exp Nutrition Therapy/ or exp "Quality of Life"/ or exp body composition/ or exp Survival/ or exp survival rate/ or exp Life Expectancy/ or exp Body Mass Index/ or exp Body Weight/ or exp sarcopenia/ or exp Muscular Atrophy/ or exp Muscle Strength/ or exp albumins/ or exp Muscle, Skeletal/ or exp cachexia/ or exp health status/ or exp Transferrin/ or exp Prealbumin/ or exp Weight Loss/ or exp Protein-Energy Malnutrition/ or exp Malnutrition/ or exp Severe Acute Malnutrition/ |
| 8 | **Keywords**  (Nutrit* Markers or chemo* toxicity or chemo* dose reductions or malnutrition or malnutrition assessment or malnutrition universal screening tool or Nutritional Risk Index or survival or survival probability or Survival time or life expectancy or Cancer Survival or BMI or body mass index or skeletal muscle mass or QOL or quality of life or Weight or sarcop*enia or muscle mass or skeletal muscle or muscle wasting or muscular atrophy or body composition or cachexia or albumin or transferrin or prealbumin OR weight*loss OR malnutrition |
| 9 | *7 OR 8* |
| 10 | *3 AND 6 AND 9* (limit 10 to humans and yr="2008 - Current") |

**Supplementary Table 2.** Search Strategy Web of Science

| **Search No.** | **Search Statement** |
| --- | --- |
| 1 | (((((((((((((TS=((duoden* NEAR/3 ("cancer*" or "tumo?r*" or "neoplasm*" or "carcinoma*" or "malignan*" or "adenocarcin*")))) OR TS=((splen* NEAR/3 (“cancer*”or “tumo?r*" or “neoplasm*” or “carcinoma*” or “malignan*” or “adenocarcin*”)))) OR TS=((gall bladder NEAR/3 (“cancer*” or “tumo?r*” or “neoplasm*” or “carcinoma*” or “malignan*” or “adenocarcin*”)))) OR TS=((hepato* NEAR/3 (“cancer*”or “tumo?r*” or “neoplasm*” or “carcinoma*” or “malignan*” or “adenocarcin*”)))) OR TS=((liver NEAR/3 (“cancer*” or “tumo?r*” or “neoplasm*” or “carcinoma*” or “malignan*” or “adenocarcin*”)))) OR TS=((bil* NEAR/3 (“cancer*” or “tumo?r*” or “neoplasm*” or “carcinoma*” or “malignan*”or “adenocarcin*”)))) OR TS=((pancrea* NEAR/3 (“cancer*” or “tumo?r*” or “neoplasm*” or “carcinoma*” or “malignan*” or “adenocarcin*”)))) OR TS=((gastric NEAR/3 (“cancer*” or “tumo?r*” or “neoplasm*” or “carcinoma*” or “malignan*” or “adenocarcin*”)))) OR TS=((stomach NEAR/3 (“cancer*”or “tumo?r*” or “neoplasm*” or “carcinoma*” or “malignan*” or “adenocarcin*”)))) OR TS=((esophag* NEAR/3 (“cancer*” or “tumo?r*” or “neoplasm*” or “carcinoma*” or “malignan*” or “adenocarcin*”)))) OR TS=((oesophag* NEAR/3 (“cancer*” or “tumo?r*” or “neoplasm*” or “carcinoma*” or “malignan*”or “adenocarcin*”)))) OR TS=((upper gastrointestinal NEAR/3 (“cancer*” or “tumo?r*” or “neoplasm*” or “carcinoma*” or “malignan*”or “adenocarcin*”)))) OR TS=(cholangiocarcinoma)) OR TS=(Oncolo*) |
| 2 | (((((((((((((((((((((TS = (jejun* NEAR/3 (“feed*” or “enteral” or “tube” or “ostomy”)))) OR (TS = (gastr* NEAR/3 (“feed*” or “enteral” or “tube” or “ostomy”)))) OR TS=(enteral nutrition)) OR TS=(enteral feeding)) OR TS=(tube feeding)) OR TS=(enteral tube feeding )) OR TS=(nutritional support )) OR TS=(enteral nutrition therapy)) OR TS=(enteral nutrition support )) OR TS=('peg' )) OR TS=(percutaneous endoscopically placed gastrostomy )) OR TS=(percutaneous endoscopic gastrostomy)) OR TS=(nasogastric feeding tube)) OR TS=(nasogastric tube)) OR TS=(nasojejunal feeding tube)) OR TS=(nasojejunal tube)) OR TS=(jejunostomy)) OR TS=(jejunostomy feeding)) OR TS=(jejunostomy feeding tube)) OR TS=(jejunal feeding)) OR TS=(gastric feeding) |
| 3 | (((((((((((((((((((((((((((((((((((((TS=(Nutrition Assessment)) OR TS=(Nutritional Status)) OR TS=(Nutrition Therapy)) OR TS=(body composition)) OR TS=(survival rate)) OR TS=(Body Mass Index)) OR TS=(Muscle Strength)) OR TS=(health status)) OR TS=(Biomarkers)) OR TS=(Nutrit* Markers)) OR TS=(chemo* toxicity)) OR TS=(chemo* dose reductions)) OR TS=(malnutrition)) OR TS=(malnutrition assessment)) OR TS=(malnutrition universal screening tool )) OR TS=(Nutritional Risk Index )) OR TS=(survival )) OR TS=(survival probability)) OR TS=(Survival time)) OR TS=(life expectancy )) OR TS=(Cancer Survival )) OR TS=(BMI )) OR TS=(skeletal muscle mass)) OR TS=(QOL)) OR TS=(quality of life)) OR TS=(sarcop*enia)) OR TS=(muscle mass)) OR TS=(skeletal muscle)) OR TS=(muscle wasting)) OR TS=(muscular atrophy)) OR TS=(body composition)) OR TS=(cachexia)) OR TS=(malnutrition)) OR TS=(Weight loss)) OR TS=(albumin)) OR TS=(transferrin)) OR TS=(prealbumin)) |
| 4 | 1 AND 2 AND 3 (limit 4 to search period: 2008-2023) |

**Supplementary Table 3.** Search Strategy Embase

| Search No. | Search Statement |
| --- | --- |
| 1 | **Medical Headings**  exp gastric metastasis/ or exp esophagus cancer/ or exp pancreas cancer/ or exp pancreas tumor/ or exp esophageal adenocarcinoma/ or exp stomach adenocarcinoma/ or stomach cancer/ or exp stomach tumor/ or exp stomach carcinoma/ or exp hepatobiliary system carcinoma/ or exp hepatobiliary system cancer/ or exp liver cell carcinoma/ or exp bile duct carcinoma/ or exp duodenum cancer/ or exp duodenum carcinoma/ |
| 2 | **Key words**  (Oncolo* or cholangiocarcinoma or (duoden* adj3 (cancer*or tumo?r* or neoplasm*or carcinoma* or malignan* or adenocarcin*)) or (hepato* adj3 (cancer*or tumo?r* or neoplasm*or carcinoma* or malignan* or adenocarcin*)) or (liver adj3 (cancer* or tumo?r* or neoplasm* or carcinoma* or malignan*or adenocarcin*)) or (bil* adj3 (cancer* or tumo?r* or neoplasm* or carcinoma* or malignan*or adenocarcin*)) or (pancrea* adj3 (cancer*or tumo?r* or neoplasm*or carcinoma* or malignan* or adenocarcin*)) or (gastric adj3 (cancer* or tumo?r* or neoplasm* or carcinoma* or malignan*or adenocarcin*)) or (stomach adj3 (cancer*or tumo?r* or neoplasm*or carcinoma* or malignan* or adenocarcin*)) or (esophag* adj3 (cancer*or tumo?r* or neoplasm*or carcinoma* or malignan* or adenocarcin*)) or (oesophag* adj3 (cancer* or tumo?r* or neoplasm* or carcinoma* or malignan*or adenocarcin*)) or (upper gastrointestinal adj3 (cancer* or tumo?r*or neoplasm* or carcinoma* or malignan*or adenocarcin*))).mp. |
| 3 | 1 OR 2 |
| 4 | **Medical headings**  exp enteric feeding/ or exp nutritional support/ or exp nutrition supplement/ or exp digestive tract intubation/ |
| 5 | **Key words**  ((jejun* adj3 (feed* or enteral or tube or ostomy)) or (gastr* adj3 (feed* or enteral or tube or ostomy)) or (duo* adj3 (feed* or enteral or tube)) or enteral nutrition or enteral feeding or tube feeding or enteral tube feeding or nutritional support or enteral nutrition therapy or enteral nutrition support or 'peg' or percutaneous endoscopically placed gastrostomy or percutaneous endoscopic gastrostomy or nasogastric feeding tube or nasogastric tube or nasojejunal feeding tube or nasojejunal tube or jejunostomy or jejunostomy feeding or jejunostomy feeding tube or jejunal feeding or gastric feeding).mp. |
| 6 | 4 OR 5 |
| 7 | **Medical Headings**  exp malnutrition assessment/ or exp malnutrition/ or exp Malnutrition Universal Screening Tool/ or nutrition/ or nutritional assessment/ or nutrient management/ or exp nutritional status/ or exp body composition/ or "quality of life"/ or survival/ or survival index/ or exp cancer survival/ or exp survival rate/ or exp survival time/ or exp life expectancy/ or exp body mass/ or exp muscle atrophy/ or exp sarcopenia/ or exp muscle mass/ or exp Nutritional Risk Index/ or albumin/ or exp body weight/ or weight/ or exp skeletal muscle/ or exp cachexia/ or exp body composition/ or exp health status/ or exp transthyretin/ or exp malnutrition/ or exp protein calorie malnutrition/ or exp body weight loss/ |
| 8 | **Keywords**  Nutrit* Markers OR chemo* toxicity OR chemo* dose reductions OR survival OR survival probability OR BMI OR body mass index OR skeletal muscle mass OR QOL OR quality of life OR Weight OR sarcop*enia OR muscle mass OR skeletal muscle OR muscle wasting OR muscular atrophy OR body composition OR cachexia OR albumin OR prealbumin OR transthyretin |
| 9 | *7 OR 8* |
| 10 | *3 AND 6 AND 9* (limit 10 to humans and yr="2008 - Current") |

**Supplementary Table 4.** Search Strategy Cochrane

| **Search No.** | **Search Statement** |
| --- | --- |
| 1 | **Medical Headings**  Esophageal Neoplasms OR Pancreatic Neoplasms OR Esophageal Neoplasm OR Stomach Neoplasms OR Liver Neoplasms OR Bile Duct Neoplasms OR Duodenal Neoplasms |
| 2 | **Key words**  Oncolo* OR cholangiocarcinoma OR (duoden* NEAR (cancer*or tumo?r* or neoplasm*or carcinoma* or malignan* or adenocarcin*)) OR (splen* NEAR (cancer*or tumo?r* or neoplasm*or carcinoma* or malignan* or adenocarcin*)) OR (gall bladder NEAR (cancer*or tumo?r* or neoplasm*or carcinoma* or malignan* or adenocarcin*)) OR (hepato* NEAR (cancer*or tumo?r* or neoplasm*or carcinoma* or malignan* or adenocarcin*)) OR (liver NEAR (cancer* or tumo?r* or neoplasm* or carcinoma* or malignan*or adenocarcin*)) OR (bil* NEAR (cancer* or tumo?r* or neoplasm* or carcinoma* or malignan*or adenocarcin*)) OR (pancrea* NEAR (cancer*or tumo?r* or neoplasm*or carcinoma* or malignan* or adenocarcin*)) OR (gastric NEAR (cancer* or tumo?r* or neoplasm* or carcinoma* or malignan*or adenocarcin*)) OR (stomach NEAR (cancer*or tumo?r* or neoplasm*or carcinoma* or malignan* or adenocarcin*)) OR (esophag* NEAR (cancer*or tumo?r* or neoplasm*or carcinoma* or malignan* or adenocarcin*)) OR (oesophag* NEAR (cancer* or tumo?r* or neoplasm* or carcinoma* or malignan*or adenocarcin*)) OR (upper gastrointestinal NEAR (cancer* or tumo?r*or neoplasm* or carcinoma* or malignan*or adenocarcin*)) |
| 3 | 1 OR 2 |
| 4 | **Medical headings**  Enteral Nutrition OR Nutritional Support OR Nutrition Assessment OR Intubation, Gastrointestinal |
| 5 | **Key words**  (jejun* NEAR (feed* or enteral or tube or ostomy)) OR (gastr* NEAR (feed* or enteral or tube or ostomy)) OR (duo* NEAR (feed* or enteral or tube)) OR enteral nutrition OR enteral feeding OR  tube feeding OR enteral tube feeding OR nutritional support OR enteral nutrition therapy OR  enteral nutrition support OR 'peg' OR percutaneous endoscopically placed gastrostomy OR percutaneous endoscopic gastrostomy OR nasogastric feeding tube OR nasogastric tube OR nasojejunal feeding tube OR nasojejunal tube OR jejunostomy OR jejunostomy feeding OR jejunostomy feeding tube OR jejunal feeding OR gastric feeding |
| 6 | 4 OR 5 |
| 7 | **Medical Headings**  Severe Acute Malnutrition OR Protein-Energy Malnutrition OR Malnutrition OR [Nutritional Status](https://www-cochranelibrary-com.ez.library.latrobe.edu.au/advanced-search/mesh?p_p_id=58_INSTANCE_MODAL&p_p_lifecycle=0&p_p_state=normal&saveLastPath=false&_58_INSTANCE_MODAL_redirect=%2Fadvanced-search%2Fmesh#0)  [Nutrition Therapy](https://www-cochranelibrary-com.ez.library.latrobe.edu.au/advanced-search/mesh?p_p_id=58_INSTANCE_MODAL&p_p_lifecycle=0&p_p_state=normal&saveLastPath=false&_58_INSTANCE_MODAL_redirect=%2Fadvanced-search%2Fmesh#0) OR [Nutrition Assessment](https://www-cochranelibrary-com.ez.library.latrobe.edu.au/advanced-search/mesh?p_p_id=58_INSTANCE_MODAL&p_p_lifecycle=0&p_p_state=normal&saveLastPath=false&_58_INSTANCE_MODAL_redirect=%2Fadvanced-search%2Fmesh#0) OR Nutritional Status OR Quality of Life OR Survival  Survival Rate OR Life Expectancy OR Body Composition OR Body Mass Index OR Body Weight  Muscular Atrophy OR Sarcopenia OR Muscle, Skeletal OR Cachexia OR Albumins OR Prealbumin  Transferrin OR Weight loss |
| 8 | **Keywords**  Nutrit* Markers OR chemo* toxicity OR chemo* dose reductions OR survival OR survival probability OR BMI OR body mass index OR skeletal muscle mass OR Malnutrition Universal Screening Tool OR Malnutrition assessment OR QOL OR quality of life OR Weight OR sarcop*enia OR muscle mass OR skeletal muscle OR muscle wasting OR muscular atrophy OR body composition OR cachexia OR albumin OR OR transferrin OR prealbumin OR malnutrition OR weight*loss |
| 9 | *7 OR 8* |
| 10 | *3 AND 6 AND 9* (limit 10 to yr="2008 - Current") |

**Supplementary Table 5.** Search Strategy CINAHL EBSCOhost

| **Search No.** | **Search Statement** |
| --- | --- |
| 1 | **Medical Headings**  (MH "Biliary Tract Neoplasms+") OR (MH "Liver Neoplasms+") OR (MH "Pancreatic Neoplasms+") OR (MH "Stomach Neoplasms+") OR (MH "Esophageal Neoplasms+") OR (MH "Duodenal Neoplasms+") OR (MH "Bile Duct Neoplasms+") OR (MH "Gallbladder Neoplasms+") |
| 2 | **Key words**  Oncolo* OR cholangiocarcinoma* OR ("upper gastrointestinal" N3 (cancer* OR tumo#r* OR neoplasm* OR carcinoma* OR malignan* OR adenocarcin*) ) OR (splen* NEAR/3 (cancer*or tumo?r* or neoplasm*or carcinoma* or malignan* or adenocarcin*)) OR (gall bladder NEAR/3 (cancer*or tumo?r* or neoplasm*or carcinoma* or malignan* or adenocarcin*))  OR (oesophag* N3 (cancer* OR tumo#r* OR neoplasm* OR carcinoma* OR malignan* OR adenocarcin*) ) OR (esophag* N3(cancer* OR tumo#r* OR neoplasm* OR carcinoma* OR malignan* OR adenocarcin*) ) OR (stomach* N3(cancer* OR tumo#r* OR neoplasm* OR carcinoma* OR malignan* OR adenocarcin*) ) OR (gastric* N3 (cancer* OR tumo#r* OR neoplasm* OR carcinoma* OR malignan* OR adenocarcin*) ) OR (pancrea* N3 (cancer*OR tumo#r* OR neoplasm* OR carcinoma* OR malignan* OR adenocarcin*) ) OR (bil* N3 (cancer* OR tumo#r* OR neoplasm*OR carcinoma* OR malignan* OR adenocarcin*) ) OR (liver N3 (cancer* OR tumo#r* OR neoplasm* OR carcinoma* OR malignan* OR adenocarcin*) ) OR  (hepato* N3 (cancer* OR tumo#r* OR neoplasm* OR carcinoma* OR malignan* OR adenocarcin*) ) OR(duoden* N3 (cancer* OR tumo#r* OR neoplasm* OR carcinoma* OR malignan* OR adenocarcin*) ) |
| 3 | 1 OR 2 |
| 4 | **Medical headings**  (MH "Enteral Nutrition+") OR (MH "Intubation, Gastrointestinal+") OR (MH “Nutritional Support+”) OR (MH “Dietary supplements+”) |
| 5 | **Key words**  (jejun* N3 (feed* or enteral or tube or ostomy)) OR (gastr* N3 (feed* or enteral or tube or ostomy)) OR (duo* N3 (feed* or enteral or tube)) OR enteral nutrition OR enteral feeding OR tube feeding OR enteral tube feeding OR nutritional support OR enteral nutrition therapy OR enteral nutrition support OR 'peg' OR percutaneous endoscopically placed gastrostomy OR percutaneous endoscopic gastrostomy OR nasogastric feeding tube OR nasogastric tube OR nasojejunal feeding tube OR nasojejunal tube OR jejunostomy OR jejunostomy feeding OR jejunostomy feeding tube OR jejunal feeding OR gastric feeding |
| 6 | 4 OR 5 |
| 7 | **Medical Headings**  (MH "Nutritional Assessment") OR (MH "Malnutrition+") OR (MH "Protein-Energy Malnutrition+") OR (MH "Nutritional Support+") OR (MH "Nutritional Status") OR (MH "Nutritional Assessment") OR (MH "Survival") OR (MH "Quality of Life+") OR (MH "Life Expectancy+") OR (MH "Body Composition+") OR (MH "Nutritional Status") OR (MH "Body Mass Index") OR (MH "Health Status Indicators") OR (MH "Muscular Atrophy+") OR (MH "Muscle Weakness+") OR (MH "Body Weights and Measures") OR (MH "Sarcopenia") OR (MH "Albumins+") or (MH "Muscle, Skeletal+")  OR (MH "Body Composition+") OR (MH "Cachexia") OR (MH "Malnutrition+") OR (MH "Nutrition Disorders+") OR (MH "Protein-Energy Malnutrition") OR (MH "Weight Loss+") OR (MH "Transferrin") |
| 8 | **Keywords**  Nutrit* Markers OR chemo* toxicity OR chemo* dose reductions OR survival OR survival probability OR BMI OR body mass index OR skeletal muscle mass OR QOL OR quality of life OR Weight OR sarcop*enia OR muscle mass OR skeletal muscle OR muscle wasting OR muscular atrophy OR body composition OR cachexia OR weight loss* OR malnutrition OR albumin OR prealbumin OR transferrin |
| 9 | *7 OR 8* |
| 10 | *3 AND 6 AND 9* (limit 10 to humans and yr="2008 - Current") |
